# From Basic Immunology to Clinical Practice: Bio-Originators versus Bio-Similars

**DOI:** 10.31138/mjr.30.1.54

**Published:** 2019-05-31

**Authors:** Stamatis-Nick C. Liossis, Georgia M. Konstantopoulou

**Affiliations:** 1 Division of Rheumatology, Department of Internal Medicine, Patras University Hospital, Rion, Patras, Greece,; 2 University of Patras Medical School, Rion, Patras, Greece

**Keywords:** Bio-Originators, biosimilars, immunogenicity, pharmacovigilance

## Abstract

Biologic agents are macromolecules, and as such, they have a high level of structural heterogeneity. Treatment with such agents has been extremely expensive limiting thus their availability to increasing numbers of patients; therefore, many manufacturers chose to develop biologics that are highly similar to the originators, the biosimilars. The immunological properties of both products should therefore be characterized and compared. The biosimilar developers must have a complete qualitative documentation, appropriate preclinical pharmacodynamic and pharmacokinetic studies, and finally comparative studies with the originator to define the relative similarity in terms of biologic activity, quality characteristics, efficacy and safety. Immunogenicity assessment of the biosimilars continues through clinical trials and pharmacovigilance programs.

## INTRODUCTION

In the past decades, the capacity to produce biologic agents ranging from growth factors and hormones to complex monoclonal antibodies and fusion proteins by using the technology of recombinant DNA, has overturned the management of inflammatory diseases, such as rheumatoid arthritis (RA), psoriasis (PsO), psoriatic arthritis (PsA), Crohn’s disease (CD), and ankylosing spondylitis (AS). ^1–3^

## DEFINITIONS

### Definition of Biologic Agents

Biologic agents are medicines in which active components are created using complex biological processes rather than chemical synthesis. Biologic medicinal products primarily include protein molecules made by living bacterial, animal, or plant cells, that have been appropriately genetically engineered.^1^ In contrast to small-molecule drugs which are synthesized using chemical methods, biologics are manufactured via complex technically processes involving multiple steps. By definition therefore, biologics are macromolecules and as such they have a high level of structural heterogeneity.^4–6^ The approaching loss of patents for a number of originator biologics has driven efforts by manufacturers to be after the evolvement of biologics that are highly similar to the originators.

### Definition of Biosimilars

A biosimilar is defined by the European Medicines Agency (EMA) as “a biological medicinal product that contains a (copy) version of the active substance of an already authorized original biological medicinal product (reference medicinal product). A biosimilar establishes similarity to the reference medicinal product in terms of quality characteristics, biological activity, safety and efficacy based on a comprehensive comparability exercise”. The bio-similar developers must have a complete qualitative documentation, appropriate preclinical pharmacodynamic and pharmacokinetic studies, and finally comparative studies with the originator to define the relative similarity in terms of biologic activity, quality characteristics, efficacy and safety.^6–9^

## BIOLOGIC AGENTS USE & SAFETY

Biologic agents, particularly monoclonal antibodies and fusion proteins containing antibody components, are important tools for the treatment of a series of inflammatory diseases including a number of rheumatic diseases. Biosimilars have made remarkable pharmaceutical progress in the field of Medicine; particularly in Haematology, Oncology, Nephrology and Rheumatology, where biologics are in routine use, and have had a very important impact on patient outcomes.^10^ The clinical use of these biologic agents, and their biosimilars, may be associated with a lot of safety issues and newly-induced though highly-unwanted immune responses. The assessment of safety, particularly immunogenicity, is very important for the biosimilar development process, similarly to the development of reference products. The routine use of biologics, including monoclonal antibodies in the rheumatological field, is therefore very important for the evolvement of biosimilars research: this is the reason of the existence of a lot of ongoing clinical trials related to rheumatology.^11^ It is very important that clinicians, before prescribing a biosimilar, understand the totality of proof supporting its biosimilarity to the reference product and the basic characteristics that a biotherapeutic must have, to be labelled a biosimilar product.^12^ The beginning of the biosimilar evolvement process is the establishment of the target quality attributes for the biosimilar. By definition, these are defined by the properties of the reference product to which the biosimilar is intended to be “highly similar”. The need for demonstrating molecular equivalence requires a panel of analytical methods, because there is no single analytical test or non-clinical/clinical study that can be sufficient to demonstrate a high level of similarity of a biosimilar with a reference biologic on its own.^13^

## THE MANUFACTURING PROCESS

Biologics are large-molecule medicines manufactured biologically, rather than chemically, using a living cell line cultured in conditions that are constantly under strict control. The first step of the biologic manufacturing process is the production of a cell line which contains the gene that makes the required protein in sufficient quantity and with the desired post-translational modifications. During the manufacturing process of a biologic agent, there are three main steps: (1) cell expansion and protein expression; (2) protein isolation and purification; and (3) formulation and drug product packaging for patient use.^14–16^ Biopharmaceuticals are large molecules that are known to be capable of triggering an immune response, known as an anti-drug antibody (ADA) response, which could be of importance if the ADA would block the active site of the biological and hence impact its effectiveness. ADAs may also favour the formation of circulating immune complexes that eventually may affect the pharmacokinetic/pharmacodynamic (PK/PD) profile of the biological drug.^17^

In addition to receptor binding analysis and functional bio-assays, bioanalytical methods are also developed and used to determine the presence of drug- induced antibodies and the quantity of a drug in biological samples. Their appliances are critical for the evaluation of pharmacology, bioavailability, bio-equivalence, PK, and toxicology studies.^18^ The immunological properties of both products should therefore be characterized and compared.^19^ It should be clear that immunogenicity assessment of the biosimilar continues through clinical trials and pharmacovigilance programs.^20,21^

## ARE BIOSIMILARS MOLECULARLY SIMILAR?

The main question for everyone who is involved in the manufacturing process of the biosimilar is: how similar is similar enough?^22^ If there are insignificant differences between the reference product and the suggested biosimilar which are noticed during the physicochemical and the *in vivo* non-clinical investigations, the clinical studies can follow a targeted approach to address residual uncertainties related to PK, PD, efficacy, immunogenicity and safety. Taking into consideration the differences between the species, investigation in animals cannot answer all the questions related to PK and PD, but it might recognise potentially important differences between the reference product and the suggested biosimilar. Despite all the differences that can be observed, the value of animal PK and PD investigations is limited, and does not cancel the necessity for the quantitative data derived from PK and PD studies in humans.^23^ Immunogenicity evaluation in animals does not always give enough and/or relevant information about the immunogenic responses to protein products in humans: this is because animals can be excessively reactive to human proteins.^23^ In animal studies, it is very difficult to arrive to definite conclusions on the comparison of immunogenicity between the proposed biosimilar and a reference product. Despite such limitations, immunogenicity evaluation in animals has a very important position during the development of a biosimilar agent, and it can give useful information about the design of the immunogenicity assessment in humans.^23^ The question of ‘how similar is similar enough’ must be always taken into consideration, and a way to answer this question is the design of proper Phase III clinical trials addressing issues on efficacy, safety and immunogenicity. Statistical methods should be assumed when defining the similarity of the proposed biosimilar to the reference product.^24^

## IMMUNOGENICITY OF BIOLOGIC AGENTS

The most important safety concern relating to biologic agents is immunogenicity.^25–28^ All biologic agents are biologically active molecules derived from living cells, and have the capacity to provoke an immune response. There are a lot of factors that can affect a product’s immunogenic potential. The presence of impurities in the final product, modifications of the structure as a result of the manufacturing process and/or the conditions of storage can increase immunogenicity. The way of administration of the biopharmaceutical can also affect immunogenicity, with intravenous administration being less immunogenic than intramuscular or subcutaneous administration.^27,28^ Factors that are strictly related to the patient are also important, such as the genetic background and HLA makeup of the patient, what type of disease is being treated and the patient’s immune condition.^29^

### Anti-Drug Antibodies (ADA) and potential effects

The importance of the emergence/presence/activity of ADAs has been previously mentioned. The biologic activity of a biosimilar may be affected if ADAs interfere with epitope bindings and/or formation of immune complexes. Monitoring for ADA concentrations may provide essential information to clinicians that can potentially improve treatment management decisions and reduce risks. The emergence and effect(s) of ADAs on the pharmacokinetics of any biosimilar is only one among the considerations to complete a biosimilars’ immunogenicity profile. There is a strong association between ADA formation and decreased clinical efficacy of several biologics/biosimilars. Enhanced immunogenicity also has the potential to increase the frequency of AEs, particularly infusion site reactions. Nevertheless, generalizations with respect to immunogenicity potential do not apply, and immunogenicity of any biologic (originator or bio-similar) needs to be characterized on “a case-by-case” basis. Use of background therapy (such as background methotrexate) and high doses of biologic agents and/or deduction treatments been shown to reduce ADA formation. Prior detection of ADA also seems to correlate with a higher incidence of immunogenicity with subsequently administered biologics.^29^ One should keep in mind that a healthy subject population could have a different immune response to any biological treatment compared to patient populations, because patients can have modified immune functions due to their disease or the necessity to receive concomitant immune-modulating medications. It is very important to take into consideration that in some cases it is the very development of ADA which can modify the PK, and thus influence the PK assessment. Therefore, PK comparability study in a less immunogenic population might be desirable. In some cases, there isn’t enough information about the safety profile of the product and for this reason such studies may not be allowed in healthy subjects. In such a scenario, PK similarity studies will have to be conducted in an appropriate patient population in which the disease status is consequent with the indication for which the proposed biosimilar is being produced.^29,31,32^

## SAFETY AND EFFICACY OF BIOSIMILARS

Finally, although cautiously, it can be expected that an approved biosimilar which shows similar safety and efficacy to a reference product in one or more indications, may also be similarly efficacious and safe in other disease situations for which the reference product is approved. Nevertheless, additional proof may be reassuring for some physicians.^32^ The above are summarized in *Table 1*.

**Table 1.** Important Aspects for the successful development of Biosimilars.

· High level of molecular similarity to originators
· Efficacy
· Safety
· Low Immunogenicity (formation of ADA)
· Feasible way of administration (i.v., i.m.,s.c.)

### The Exchangeability Issue

Exchangeability and automatic replacement between the reference product and the biosimilar are actively being discussed; this is why the decision of a rheumatologist to replace a biologic agent with a biosimilar is not always straightforward. Attentive pharmacovigilance is necessary to collect clinical data with respect to short- and long-term safety of biosimilars. Being highly similar in structure to the reference product, the clinician should expect comparable efficacy and safety, ideally for a reduced cost and the patient must not be affected in an unfavourable way.^33^ At the end, it is important to discuss which should be the role of the patients in the plans that refer to their treatment. They must be able to receive all the information they need about their therapeutic process. However, it is likely that they will be decisively influenced by physician guidance concerning use of biosimilars and they may not be aware whether they are being treated with a biosimilar or an originator product. Society thus faces two important health care paradigms: patient participation in their health care plans, and use of biosimilars.^34^

## CONCLUSION

A biosimilar and its originator contain basically the same active biological, though there may be minor differences due to their complex nature and production methods.^35^ The main concern during the development of a biosimilar is the establishment of high similarity based on expansive comparability analytical assessments.^26^ Biosimilars must undergo an accurate development process to demonstrate, using the “totality of evidence” that any eventual differences will not influence the clinical performance of the product, compared with the reference product.^37^ If the comparative and physicochemical studies conducted *in vitro*, demonstrate a high level of similarity, is less likely that new safety issues will arise.^38^ However, one should always take into consideration that these biomolecules are so complex and even minor differences may potentially seriously influence their characteristics and properties.
